# Time elapsed since peruvian children’s last dental care and head of household educational attainment: findings from a national database

**DOI:** 10.1186/s12903-023-03083-y

**Published:** 2023-06-09

**Authors:** María Claudia Garcés-Elías, César Eduardo Del Castillo-López, Jorge A. Beltrán, Roberto A. León-Manco

**Affiliations:** Facultad de Estomatología, Universidad Peruana Cayetano Heredia, Av. Honorio Delgado 430, San Martín de Porres, Lima, 15102 Peru

**Keywords:** Educational status, Oral health, Health services accessibility, Peru

## Abstract

**Background:**

It has been documented that the parents’ highest level of education has an impact on their children’s access to oral health services and the frequency of their use.This study aimed to determine the association between time elapsed since peruvian children’s last dental care and head of household educational attainment.

**Methods:**

Cross-sectional study using a database of children aged 0 to 11 years, with a final sample of 8012 participants. The dependent variable in this study was the time elapsed since last dental care and the independent variable was the head of household educational attainment. Other covariates considered were natural region, area of residence, place of residence, altitude, wealth index, health insurance coverage, sex and age. Descriptive, bivariate and multivariate statistical analyses were applied.

**Results:**

Time elapsed since last dental care in the year 2021 was 5.68 years (SD = 5.25). A hierarchical multiple linear regression analysis was performed, analyzing the variables dimensions by separate and joint models. When head of household educational attainment was analyzed, there was no statistical significance (p = 0.262); however, other models did (p < 0.05). Model 4, which addresses all dimensions, was significant (p < 0.001) with an R^2^% of 0.011 and constant equal to 5.788; it showed significance with place of dental care, health insurance, altitude and age.

**Conclusions:**

No association was found between head of household educational attainment and time elapsed since last dental care; however, the latter was associated with place of care, health insurance coverage, altitude and age in Peruvian children.

## Background

Access to dental care refers to the ability of individuals to make timely use of oral health services for early diagnosis, prevention and restorative treatment in order to achieve the best possible outcomes. This concept includes physical, financial and user acceptability dimensions [1, 2]. Considering this, the World Health Organization (WHO) describes that access to dental health care services on an international scale is at a critical level, characterized by the presence of considerable and constant inequalities. The inadequate provision and utilization of dental health services can affect the self-perceived need for treatment, which is an indicator for estimating dissatisfaction with access to care in oral health services [3].

This situation could be associated with factors inherent to health systems, such as the scarcity of human resources and their inequitable distribution, as well as the limited presence of adequate health infrastructure to meet the needs of the community. Likewise, out-of-pocket payment stands out as one of the main barriers to the use of dental health services, considering that access to this type of care in a considerable number of countries is not guaranteed to the entire population and has coverage limitations [4].

On the other hand, the association between social determinants of health and use of dental services has been documented, with the implication of the level of education on oral health conditions and the exercise of healthy practices that favor it standing out [5–7]. In this sense, it is observed that the highest level of education of parents has an impact on their children’s access to oral health services and the frequency of their use [8], which is supported by findings that link the mother’s education as a preponderant predictor of conditions that affect the oral cavity of her children [9]. Internationally, the oral health inequities that children face are persistent, especially in low- and middle-income countries where the coverage, availability and access to oral health services is mediated by economic, geographic and educational characteristics of their families [10]; however, the evidence documenting the aforementioned problem within a national context is scarce. Consequently, the aim of this research was to determine the association between time elapsed since peruvian children’s last dental care and head of household educational attainment during 2021.

## Methods

### Design and settings

This manuscript was written in accordance with the STROBE initiative for observational studies. A cross-sectional study was developed, using a database of the Demographic and Family Health Survey (ENDES) for year 2021, applied annually through home interviews by the National Institute of Statistics and Informatics (INEI). The survey has a two-stage cluster sampling design, probabilistic, balanced, stratified and independent, representative at the national and regional levels, and according to urban and rural areas. For the 2021 sample frame, the ENDES information is derived from the 2017 National Population Census XII and Housing Census VII and the Household Targeting System of 2012–2013. Regarding the departmental distribution by rural and urban area, a sample size of 36,760 dwellings was obtained, which were distributed in 14,780 dwellings in departmental capitals and districts of the province of Lima, 9320 dwellings in the remaining urban area and 12,660 rural dwellings. In addition, this survey provides information on the time elapsed since last dental care for children between 0 and 11 years old. It should be mentioned that the complete records referring to time since last dental care were 42,115, however, only those that included the educational attainment of the head of household were considered, defining a final sample size of 8012 records. It should be mentioned that ENDES is applied to all members of the household who are habitual residents.

### Variables

In this study, the dependent variable was the time elapsed since the last dental care measured in years. While for the organization of the independent variables, dimensions were generated for statistical analysis: the first was head of household educational attainment, the second addressed health characteristics including insurance and place of dental care, taking into account that in Peru there are different health providers such as the Ministry of Health (MINSA), Social Security of Peru (EsSalud), Armed Forces and Police (FF.AA./PNP) Health Insurance and the private sector [11]. The third dimension considered the geographic characteristics comprising the natural region, conceptualized as Metropolitan Lima (capital of Peru), the rest of the coast, highlands and jungle; area of residence, categorized into urban and rural; place of residence organized into capital, city, town and countryside; altitude organized into less than 2500 meters above mean sea level (MAMSL) and from 2500 MAMSL and above. Altitude is a relevant variable in high Andean countries like Peru, because it is common to have capitals or urban areas in very high areas. The last dimension covered sociodemographic characteristics such as wealth index, a variable that includes the disposition that each household has for the consumption and use of goods and services, allowing to classify each household in five quintiles (from the poorest to the richest) [12, 13]; likewise, sex and age classified in 0 to 5 years old and 6 to 11 years old; it is important to mention that the period from 0 to 5 years counts 6 years in total because zero is an additional period of time.

Likewise, the ENDES databases were extracted from the National Platform of Open Data (https://www.datosabiertos.gob.pe/dataset/encuesta-demográfica-y-de-salud-familiar-endes-2021-instituto-nacional-de-estad%C3%ADstica-e-3) through a variety of information sources that were then grouped into a single matrix, to be later analyzed in STATA v. 17 software. It should be clarified that the data analysis was carried out considering representative estimates because the sample design was included in which the sampling patterns were differentiated by stratum, primary sampling unit and weights; this is because it is a national survey and allows for establishing representative estimates.

### Statistical analysis

A descriptive analysis of the qualitative variables was carried out by means of absolute and relative frequencies, while the mean, standard deviation, median, Q1 and Q2 of the variable time elapsed since last dental care was obtained. In addition, the normality of the distribution of the dependent variable was evaluated according to the other variables using the Kolmogorov-Smirnov test; subsequently, the nonparametric Mann-Whitney U tests were applied for dichotomous variables, Kruskal-Wallis for polytomous variables and Mann-Whitney U for post hoc analysis. Next, a hierarchical multiple linear regression was developed with the purpose of generating models between the independent variables and the time of last dental care; it is important to mention that a logarithmic transformation was previously applied to the dependent variable due to the lack of a normal distribution. The reference category for the analysis is the first option considering them on an ordinal scale in each variable. The confidence level in the study was 95%, and as an indicator of statistical significance a p < 0.05 was considered in all tests.

### Ethics

The authors used freely available information provided by INEI to conduct this study, since it is a secondary analysis of anonymous information, the approval of an ethics committee is not required.

## Results

Time elapsed since last dental care in the year 2021 was 5.68 years (SD = 5.25); according to head of household educational attainment, children whose parents did not have any level of education had a time of 5.81 (SD = 5.02), for primary level 5.46 years (SD = 4.95), for secondary level 5.80 years (SD = 5.04) and for higher level 5.77 years (SD = 5.14); however, there was no statistically significant difference (p = 0.165). On the other hand, time elapsed since last dental care had statistically significant differences with place of dental care, health insurance, natural region, area of residence, place of residence, altitude and wealth index (p < 0.05) (Table 1).

**Table 1:** Association between time elapsed since peruvian children’s last dental care and head of household educational attainment

Variables			n	%				Time elapsed since last dental care			
					X	SD	Median		Q1	Q3	p
Total			8012	100.00	5.68	5.25	3.00		2.00	12.00	
Head of household educational attainment											
	None		131	1.46	5.81	5.02	3.00		2.00	12.00	0.165*
	Primary		1467	16.87	5.46	4.95	3.00		2.00	9.00	
	Secondary		3716	47.55	5.80	5.04	3.00		2.00	12.00	
	Superior		2698	34.12	5.77	5.14	3.00		2.00	12.00	
Health characteristics											
	Place of dental care										
		Ministry of Health	4877	46.49	5.84ab	5.05	3.00		2.00	12.00	< 0.001*
		Social security (EsSalud)	843	12.10	6.77ac	4.95	3.00		2.00	12.00	
		Armed forces / PNP	18	0.54	4.74	4.72	3.00		2.00	4.00	
		Private sector	2273	40.87	5.09bc	5.04	3.00		2.00	7.00	
	Health insurance coverage										
		Yes	6527	80.44	5.67	5.06	3.00		2.00	11.00	**0.025** ^†^
		No	1485	19.56	5.99	5.04	3.00		2.00	12.00	
Geographic characteristics											
	Natural region										
		Metropolitan Lima	1083	35.57	5.61a	4.91	3.00		2.00	10.00	< 0.001*
		Rest of the coast	2238	25.30	6.09ab	5.16	3.00		2.00	12.00	
		Highlands	2853	25.59	5.44bc	5.09	3.00		2.00	11.00	
		Jungle	1838	13.54	5.80c	4.94	3.00		2.00	10.00	
	Area of residence										
		Urban	5488	80.99	5.85	5.10	3.00		2.00	12.00	**0.001** ^†^
		Rural	2524	19.01	5.46	4.95	3.00		2.00	10.00	
	Place of residence										
		Capital	1083	35.58	5.61a	4.91	3.00		2.00	10.00	0.003*
		City	2326	21.62	5.96ab	5.19	3.00		2.00	12.00	
		Town	2079	23.79	5.84	5.09	3.00		2.00	12.00	
		Countryside	2524	19.01	5.46b	4.95	3.00		2.00	10.00	
	Altitude										
		Less than 2500 MAMSL	5564	78.47	5.88	5.06	3.00		2.00	12.00	**< 0.001** ^†^
		From 2500 MAMSL and above	2448	21.53	5.37	5.04	3.00		2.00	10.00	
Sociodemographic characteristics											
	Wealth index										
		Very poor	2034	16.05	5.47a	4.94	3.00		2.00	10.00	< 0.001*
		Poor	2071	21.28	5.81	5.01	3.00		2.00	12.00	
		Medium	1579	22.43	5.78b	5.10	3.00		2.00	12.00	
		Rich	1310	21.09	6.23abc	5.23	3.00		2.00	12.00	
		Very rich	1018	19.15	5.42c	5.17	3.00		2.00	10.00	
	Sex										
		Man	5640	69.23	5.74	5.06	3.00		2.00	12.00	0.723^†^
		Woman	2372	30.77	5.69	5.06	3.00		2.00	10.50	
	Age										
		From 0 to 5 years old	5766	40.23	5.68	5.25	3.00		2.00	12.00	0.226^†^
		From 6 to 11 years old	2246	59.77	5.84	4.51	3.00		2.00	8.00	

*Kruskall Wallis Test and Mann-Whitney U post hoc analysis. Between equal letters there is a statistically significant difference

^†^Mann Whitney U test

Next, a hierarchical multiple linear regression analysis was performed, analyzing the variables dimensions by separate models and as a whole. When the analysis was developed with head of household educational attainment, there was no statistical significance (p = 0.262); however, the other models were significant (p < 0.05). Model 4, which addresses all dimensions, was significant (p < 0.001) with an R^2^% of 0.011 and constant equal to 5.788; it presented significance with place of dental care (unstandardized coefficient=-0.363, 95%CI= -0.459- -0.267, p < 0.001), health insurance (unstandardized coefficient = 0.313, CI95%=0.018–0.607, p = 0.037), altitude (unstandardized coefficient=-0.423, CI95%= -0.693- -0.153, p = 0.002) and age (unstandardized coefficient = 0.378, CI95%= 0.115–0.641, p = 0.002) (Table 2).

**Table 2:** Hierarchical multiple regression model of head of household educational attainment and time elapsed since peruvian children’s last dental care

Variables			R^2^	Change of R^2^	p (Change of R^2^)	Constant	Unstandardized coefficients	Standardized coefficients	95%CI	Sig.	Model Sig.
Model 1											
	Head of household educational attainment		0.000	0.000	0.262	5.467	0.087	0.013	-0.065-0.239	0.262	0.262
Model 2											
	Head of household educational attainment		0.006	0.006	< 0.001	5.243	0.205	0.030	0.049–0.361	0.010	< 0.001
	Health characteristics										
		Place of dental care					-0.295	-0.077	-0.384- -0.206	< 0.001	
		Health insurance coverage					0.361	0.028	0.068–0.655	0.016	
Model 3											
	Head of household educational attainment		0.009	0.003	< 0.001	6.531	0.140	0.021	-0.022-0.302	0.089	< 0.001
	Health characteristics										
		Place of dental care					-0.320	-0.083	-0.410- -0.229	< 0.001	
		Health insurance coverage					0.308	0.024	0.014–0.602	0.040	
	Geographic characteristics										
		Natural region					-0.002	0.000	-0.145-0.141	0.974	
		Area of residence					-0.425	-0.039	-0.857-0.006	0.053	
		Place of residence					0.072	0.015	-0.145-0.288	0.517	
		Altitude					-0.464	-0.042	-0.731- -0.197	0.001	
Model 4											
	Head of household educational attainment		0.011	0.002	0.016	5.788	0.124	0.018	-0.046-0.295	0.153	< 0.001
	Health characteristics										
		Place of dental care					**-0.363**	**-0.094**	**-0.459- -0.267**	**< 0.001**	
		Health insurance coverage					**0.313**	**0.024**	**0.018–0.607**	**0.037**	
	Geographic characteristics										
		Natural region					0.024	0.005	-0.124-0.171	0.752	
		Area of residence					-0.380	-0.035	-0.827-0.067	0.095	
		Place of residence					0.085	0.017	-0.133-0.302	0.446	
		Altitude					**-0.423**	**-0.038**	**-0.693- -0.153**	**0.002**	
	Sociodemographic characteristics										
		Wealth index					0.101	0.027	-0.029-0.230	0.128	
		Sex					-0.069	-0.006	-0.319-0.181	0.588	
		Age					**0.378**	**0.033**	**0.115–0.641**	**0.005**	

## Discussion

Parents play a preponderant role in the adoption and reinforcement of behaviors that favor their children’s oral health, including seeking timely dental treatment [14]. Within family organization, denomination of head of household becomes important, which is linked to family decision making, including the differentiation and designation of tasks associated with authority structures within the family [15]. In Peru, this function is established considering the economic contribution to the family unit, and it is also the other household members who decide who will be the head of it. According to the latest census studies in the country, nearly two thirds of Peruvian families have a male head of household [16].

Among the findings of this study, the absence of an association between the head of household educational attainment and the time elapsed since last dental care in children residing in Peru stands out. On the contrary, Azañedo et al. previously reported that children in Peru whose caregivers had a higher level of education were more likely to have access to dental care in the last 6 months of the ENDES survey [17]. On the other hand, when evaluating factors associated with the use of dental health services in older adults, it was determined that the level of education in this population group does not increase the possibility of having received care in the last six months [18].

Likewise, the COVID-19 pandemic produced great changes in the countries, triggering critical scenarios at the health, economic and social levels; the case of Peru stands out, as it was one of the nations with a high number of infections and presented the highest case fatality rate at the international level [19, 20], requiring the implementation of drastic measures such as quarantines, social immobilization, adjustments in economic public policies, etc. [21]. During this health emergency, the use of non-COVID-19 health services has declined across the board, especially in low- and middle-income countries [22]. From a dental perspective, access to these services has also been impaired, compromising the timely utilization of oral health services in Peruvian children [23].

It should be noted that this finding is not accompanied by the presence of sociodemographic characteristics that are statistically significant and are linked to the decrease in access, indicating that the main factor affecting timely care was the pandemic context. However, within this research, an absent association is detected between the variable head of household educational attainment and the time elapsed since last dental care, with the understanding that, for this period of time, other sociodemographic factors may have more relevance.

Inside the Peruvian context, education system is divided into three levels: initial, primary and secondary education, with the understanding that its organization corresponds to the stages of human development. In addition, being a country with an important geographical diversity, there are teaching variants that are adapted to these environments, such as multigrade, alternating, tutorial and residence modalities. It is important to consider Intercultural Bilingual Education, which proposes teaching using native languages and where cultural heritage predominates. At the national level, inequalities persist in access to education and school attendance, where the poorest individuals, from rural regions and those farthest from the capital, are the ones with the most discouraging figures in terms of school dropout; where the educational level of the parents is an important factor in analyzing this last aspect. In addition, gender also plays an important role in this phenomenon, reflecting the fact that women have lower levels of school attendance, which leads to low school completion rates [24].

Respect to education in Peru during the COVID-19 pandemic, government developed various strategies focused on continuing basic education activities, which were centered on implementing teaching systems based on mass and digital media to teach classes nationwide. This denotes the emergence of efforts to protect and guarantee this right during the COVID-19 crisis, however, the expected results were not achieved, with an increase in gaps according to socioeconomic level, gender and connectivity [25].

Throughout the pandemic, health and economic sectors of the country were extremely affected, despite multiple attempts to preserve health of the Peruvian population and to establish economic, fiscal and financial policies to contain the country’s recession; it appears that at the beginning of the pandemic, provisions implemented allowed controlling the number of infections in the population, however, massive loss of human lives and the socioeconomic cost that arose later, revealed that the pandemic added to the structural characteristics of Peru foreshadowed a complex scenario [26]. The implementation of measures such as quarantine and social immobilization were not successful, mainly due to socioeconomic factors such as the supremacy of an informal economy, abrupt cut in income for many households, housing conditions with little access to basic commodities, and the precariousness of the health system. Therefore, it can be understood that economic stability is a preponderant factor in Peruvian households and was weakened in most of them during the pandemic context, and may have a greater influence than the head of household educational attainment when seeking dental care.

On the other hand, findings of this research expose the presence of more relevant variables in the timely use of oral health services in Peruvian children, highlighting that those who lack health insurance delay their time of care more. In addition, individuals under twelve years old who reside above 2500 MAMSL receive more prompt attention than those who live at lower altitudes, while children between six and eleven years old receive more delayed attention than younger Peruvian children. It is important to mention that previous studies that applied the same methodology agree that the type of health insurance is a factor that reveals inequity in access to dental care; in addition, it is mentioned that Seguro Integral de Salud (translated as Comprehensive Health Insurance)(SIS) has the largest number of beneficiaries in the country, who are treated in the facilities of the Peruvian Ministry of Health [17, 18]. On the contrary, this study observed that patients attended in the latter are those who take the longest time to receive dental care.

Among other characteristics that are associated with the time elapsed since the last dental care, it is clear from this study that the lower the altitude, the longer it will take individuals to visit a dental office. However, Calderón et al. reported that in Peruvian high Andean communities, one of the main health needs was lack of access to timely screening treatments, especially for noncommunicable diseases [27]. It should be noted that the most prevalent pathologies of the oral cavity are found within this group. Likewise, in a study that sought to understand the behavior during search for dental services in pregnant women in an upper community in Nepal, it was observed that search for dental treatment tends to be delayed, especially when specialized human resources are scarce; in addition, the nearest qualified dentists were located in districts at a distance of 80 to 100 km, where time to travel to these locations was up to 24 h [28].

On the other hand, this research determined that age was a factor associated with time it takes Peruvian children to obtain dental care. In this regard, Soares et al. point out that availability of dental services in the Brazilian public sector is of concern, specifically for younger children. Despite the fact that national surveys report a 10% improvement in access to dental care, when compared to figures from the previous decade. It should be noted that more than half of children up to 6 years old had never received dental care, and even though their mothers have a high level of education, only 40% of their children have visited a dentist [29].

One of the limitations of the present study is the use of national databases, which could have been subject to information bias during data collection. In addition, the cross- sectional design of the study does not have the capacity to infer causal relationships. Furthermore, scientific evidence defining the terms “head of the household” have been scarce, so it is suggested that documents be created to standardize a definition.

Head of household educational attainment is a determining factor and has a great impact on lives of their children or caregivers, especially on their health and quality of life, which involves adequate access to and utilization of oral health services. However, in complex scenarios such as the COVID-19 pandemic, this situation seems to have changed and aggravated certain inequities related to purchasing power and economic stability, leaving in second place the professional training and maximum educational attainment achieved by parents or caregivers, which prior to the pandemic could have guaranteed financial security and enjoyment of basic services.

## Conclusions

This study can conclude that no association was found between head of household educational attainment and time elapsed since last dental care in Peruvian children; however, some specific factors like place of care, health insurance coverage, altitude and age showed association.

## Data Availability

The 2021 dataset analysed during the current study are available in the National Institute of Statistics and Informatics of Perú repository section – National Platform of Open data, (https://www.datosabiertos.gob.pe/dataset/encuesta-demográfica-y-de-salud-familiar-endes-2021-instituto-nacional-de-estad%C3%ADstica-e-3).

## Abbreviations

WHO: World Health Organization
ENDES: Demographic and Family Health Survey
INEI: National Institute of Statistics and Informatics
MINSA: Ministry of Health
EsSalud: Social Security of Peru
FF.AA./PNP: Armed Forces and Police
MAMSL: Meters above mean sea level
SD: Standard deviation
SIS: Comprehensive Health Insurance
95%CI: 95% confidence interval
